# Paper-Based Laminates Impregnated with a Hybrid Lignin-Phenol-Formaldehyde Resin

**DOI:** 10.3390/ma16072669

**Published:** 2023-03-27

**Authors:** Miroslav Němec, Kateřina Hájková, Štěpán Hýsek

**Affiliations:** Department of Wood Processing and Biomaterials, Faculty of Forestry and Wood Sciences, Czech University of Life Science Prague, Kamýcká 129, 165 21 Prague, Czech Republic

**Keywords:** kraft lignin, laminate, phenol formaldehyde, pressing, mechanical properties

## Abstract

In this study, high-pressure laminates (HPL) impregnated with phenol-formaldehyde (PF) resins enriched with kraft lignin were developed. Pulverised kraft lignin was added to the commercial PF resin in the amounts of 1% and 5% (solid to solid). Laminates were manufactured using pressure impregnation of the resins into the papers and using hot pressing of HPL in a laboratory press. Laminates with a lignin content of 1% (L-LPF-1) showed the highest bending strength (72.42 MPa) and Brinell hardness (9.41); they also exhibited the best moisture uptake (9.61) and thickness swelling after immersion in water (3.32%). Except for impact bending, laminates with a lignin content of 5% (L-LPF-5) had worse properties. However, the differences between the variants are mostly not statistically significant and are comparable with the results of commercial PF resin. Scanning electron microscopy confirmed the homogenous structure of produced laminates and the occurrence of cohesive failures in ruptured L-LPF-1 laminates, whereas in ruptured L-LPF-5 laminates adhesive failures were also observed. Based on the conducted research it can be said that the utilisation of kraft lignin as an additive to PF resin (in the amount of 1%) has a positive effect on the produced HPL.

## 1. Introduction

Paper-based laminates are made from sheets of paper impregnated with a thermosetting resin and can be classified according to their purpose for decorative or structural applications. In construction, they can be used for claddings, and various design facades can be created from HPL [1]. HPL production is an important application for phenol-formaldehyde (PF) resins [2]. PFs are synthetic polymers obtained using the reaction of phenol (P) and formaldehyde (F) [3]. PF resins are classified as synthetic adhesives, which means that the raw material for their production comes from petrochemical products [4]. The price of phenol depends on the price of oil and is likely to increase over time due to the scarcity of fossil resources [5]. A characteristic that makes PF resins invaluable is their ability to provide a composite with resistance to water, weathering, and high temperatures at a relatively low cost [6,7].

**Figure 1:**
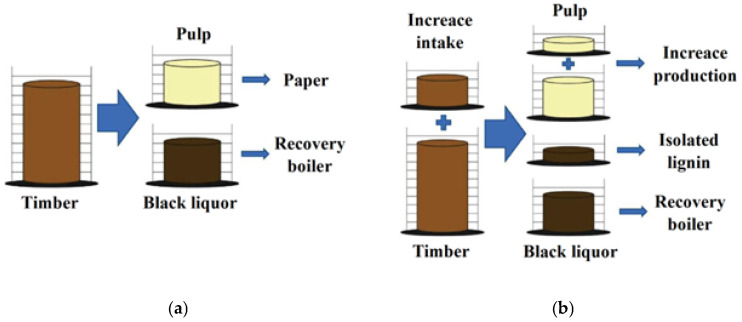
Diagram of the current state of paper industry production (**a**); and benefits of the isolation of lignin for other industrial uses (**b**).

Together with cellulose and hemicelluloses, lignin is a basic component of plants; thus, it is a widespread polymer with a phenolic nature, which makes lignin a suitable substitute in wood bonding resins [8]. Lignin is considered a waste or by-product of the paper industry and biorefineries for the production of ethanol from cellulose. The majority of lignin produced in this way is used in these plants for combustion in regeneration boilers for the production of heat and electricity (Figure 1), and a small amount of lignin is used as fillers, for example for ink varnishes, paints, and elastomeric matrices or surfactants and dispersants [9]. Due to its costs, lignin is a competitive input material. However, lignin from biomass and by-products of the paper industry is still underutilised [10].

Lignin shows relatively stable prices on the market, with prices ranging from EUR 40 to 245 EUR/MT for low purity lignin, while high-purity lignin fetches up to 655 EUR/MT. Sulphur-containing kraft lignin is on the market with a price of 225–440 EUR/MT [11]. The significantly lower price has convinced the wood glue industry to step up their efforts to replace phenol with lignin at a commercial level [2]. Phenol is highly toxic, and the International Agency for Research on Cancer (IARC; advisory committee of the World Health Organization WHO) ranks formaldehyde in group 1 from the point of view of carcinogenic effects for humans—so it is a human carcinogen [12]. Lignin is able to bind the emission of formaldehyde and thereby enhance the ecological properties of adhesives [10].

The disadvantage of lignin is its low reactivity. Lignin can be used as a filler and can replace <20–30 wt. % of some reactive components in polymers (such as phenols for phenolic resins or Bisphenol-A for epoxy resins) [9]. To increase the reactivity of lignin, it is possible to modify it, where the modification of the lignin structure consists of increasing the reactivity of hydroxyl groups [11].

The utilisation of lignin-phenol-formaldehyde (LPF) resins in the production of plywood, particleboard, or fibreboard has already been investigated. The possibility of using lignin in the production of high-pressure laminates has previously been investigated [2,13,14,15,16]. In the above-mentioned cases, a proprietary lignin-phenol-formaldehyde resin was always synthesised, where a certain proportion of phenol was replaced with unmodified or modified lignin. However, the technologically relatively simple and economically advantageous method of using kraft lignin as a filler in PF resins for the production of HPL has not been addressed in the research to date.

The goal of this study is to monitor the effect of lignin as a filler in PF for the production of HPL. Subsequently, the effect of this treatment (which can be relatively simply implemented industrially) on the properties of the produced resin was evaluated as well as the effect on the production and properties of the produced laminates. The novelty of this paper lies in the utilisation of pulverised lignin for adhesive modification. Our concept is to develop a method of hybrid LPF resin manufacturing that can be easily adopted industrially.

## 2. Materials and Methods

### 2.1. Materials

Advantage MF Boost kraft paper intended for decorative, compact, and other laminate applications with a basis weight of 120 g·m^−2^ (Mondi Group, Neusiedler, Austria) was used to produce high-pressure laminates. The PF resin Prefere 70 5703L (Prefere Resins, Erkner, Germany), intended for industrial impregnation of paper sheets for HPL production, was used for impregnation.

For the synthesis of LPF resin, pulverised kraft lignin was used from hardwood from the paper industry (Mondi Group; CAS: 8068-05-1). This kraft lignin was obtained after precipitation and washing with water/slightly acidic medium. Its pH was 4. The precipitated lignin was pulverised using a ball mill and the particle size was lower than 100 µm.

### 2.2. Adhesive Preparation

The commercial PF resin Prefere 70 5703L was used to produce LPF resins, to which 1% and 5% of kraft lignin (dry matter/dry matter) was added by mixing the pulverised lignin with the PF resin. The dry matter of the PF resin was determined using an MB23 moisture analyser (Ohaus, NJ, USA) and was 79.7%.

The production of lignin-phenol-formaldehyde resins took place at an elevated temperature of 65 °C and with constant stirring using a magnetic dipole. After mixing the desired amount of lignin, the mixture was stirred at an elevated temperature for another 30 min to completely mix the resins. The variants of resins produced are listed in Table 1.

### 2.3. HPL Manufacturing

Three variants (based on three variants of resins) of paper-based laminates were produced. Thirty sheets of A4 format paper (210 × 297 mm) with a basis weight of 120 g·m^−2^ were used for the production of all of them.

Pressure impregnation was applied to impregnate the paper sheets. The individual sheets of paper were evenly covered with resin and, after applying the resin to the required series (30 sheets), the papers in the impregnation container were inserted into a VTIZ 0.5 × 2 pressure impregnation chamber (VYVOS, Uherský Brod, Czech Republic), where a pressure of 0.5 MPa was applied. After 15 min, the pressure in the impregnation chamber was equalised and the container with the paper sheets was removed.

**Figure 2:**
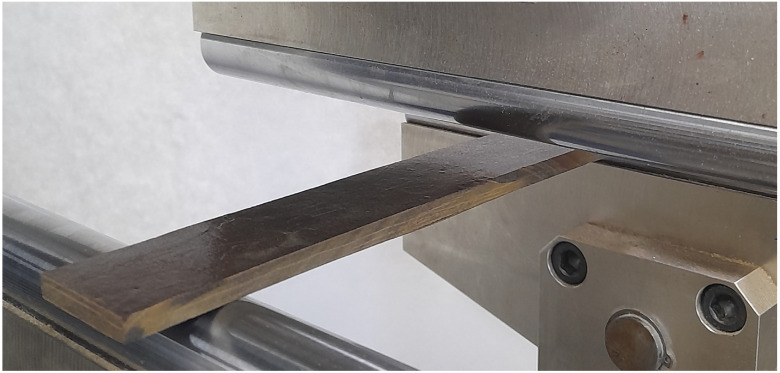
Manufactured sample during the bending test.

After impregnation, excess resin was removed using a prepared fixture (consisting of an inclined fixed plate and a moving cylinder) that simulated real industrial production. Individual sheets of impregnated paper were placed on racks on which they were placed into the dryer. The drying parameters were set to 70 °C for 60 min. After the drying step, the paper sheets were cooled at room temperature for 60 min. After cooling, the sheets of paper were stacked on top of each other and the stacked set was moved to an RLB 45-8 heated press (TOS Rakovník, Rakovnik, Czech Republic). The pressing parameters were as follows: pressing temperature 160 °C, pressure 7.215 MPa, and pressing time 20 min. After pressing, the finished laminate was removed from the press and kept under load between two steel plates for 20 min to cool down and prevent unwanted deformation of the laminates. The manufactured sample during the bending test is shown in Figure 2.

### 2.4. Methodology of Experiments

Advantage MF Boost kraft paper intended for decorative, compact, and other laminate applications with a basis weight of 120 g·m-2 (Mondi Group) was used to produce high-pressure laminates. The PF resin Prefere 70 5703L (Prefere Resins, Erkner, Germany), intended for industrial impregnation of paper sheets for HPL production, was used for impregnation.

#### 2.4.1. Fourier-Transform Infrared Spectroscopy

Fourier-transform infrared spectroscopy (FTIR) was analysed in ATR mode using a Nicolet iS20 spectroscope (Nicolet CZ, Prague, Czech Republic). Spectra were obtained with a resolution of 4 cm^−1^. Each of the resins was tested in both uncured and cured forms. Curing of the resins for FTIR analysis took place in a glass container in a drying chamber at a temperature of 103 °C for 48 h.

#### 2.4.2. Chemical Analysis of Resins

The prepared resins were analysed using a DR 6000UV-VIS spectrophotometer (Hach, Loveland, CO, USA). Specifically, the amount of phenol, tannin, and formaldehyde was analysed. Since this is a type of spectrophotometer for liquid samples, uncured resins were analysed. The samples had to be diluted due to their high absorbance; methanol was used as the diluting liquid. Furthermore, the general properties of the resins were determined—pH value, density, and total solids of the resins.

#### 2.4.3. Moisture Resistance of Laminates

Moisture resistance was measured on 10 samples (40 × 40 × 7 mm) from each variant. The test was carried out according to the EN 438-2 [17] standard. Absolute 0% moisture content of the samples was achieved by drying them in an oven at 103 °C after measuring the dimensions and weight. The samples were immersed in a water bath at a temperature of 65 °C for 48 h. After this time, the samples were removed and immersed in a container of distilled water at a temperature of 23 °C for 15 min. Thickness swelling in % (TS) and moisture uptake in % (MU) were calculated as follows:

(1) $$\mathrm{TS}=\frac{t_{w}-t_{0}}{t_{0}},$$

where t_w_ is the thickness of samples after immersion in water for 48 h in mm, and t_0_ is the thickness of the samples at 0% absolute moisture in mm.

(2) $$\mathrm{MU}=\frac{m_{w}-m_{0}}{m_{0}},$$

where m_w_ is the weight of samples after immersion in water for 48 h, and m_0_ is the weight of samples at 0% absolute moisture in g.

#### 2.4.4. Vertical Density Profile

Measurement of the vertical density profile (VDP) was performed on four samples (50 × 10 × 7 mm) from each variant of the produced laminates. The measurement was carried out on a DPX300-LTE X-ray density analyser (IMAL PAL GROUP, San Damaso, Italy). The individual measurements were averaged to produce the resulting VDP curve.

#### 2.4.5. Scanning Electron Microscopy (SEM)

Breached HPL samples after bending tests were observed using SEM. Selected samples were sputtered with gold using a Q150R rotary coater (Quorum, Laughton, UK) and analysed using a MIRA 3 scanning electron microscope (Tescan Orsay Holding, Brno, Czech Republic). Secondary electron detector (SE) was employed; the acceleration voltage was set to 10 kV.

#### 2.4.6. Tensile Shear Strength of the Glued Joint

For the samples, according to standard EN 205 [18], radial beech wood lamellas with a thickness of 5 mm were selected. The beech wood was scanned using X-ray on a WoodEye machine (Microtec, Bressanone, Italy), where lamellas without defects were selected and subsequently formatted on a saw to the required length, and the required thickness of 5 mm was achieved using a milling cutter. These lamellas were glued with reference PF resin and with developed lignin-based resin. Curing of the resins was carried out in an RLB 45-8 press (TOS Rakovník, Rakovnik, Czech Republic) at a temperature of 160 °C and a pressure of 6.5 MPa for 15 min. After pressing, the lamellas were cut for test samples (10 × 20 × 150 mm). Nine test samples were tested from each variant.

The tensile shear strength test (TSS) of the glued joint was measured on a TT 2850 universal testing machine (TIRA, Schalkau, Germany). Tensile shear strength in MPa was determined as follows:

(3) $$\mathrm{TSS}=\frac{{F\;}_{\max}}{b\cdot l},$$

where F_max_ is the maximum force achieved on the sample in N; b and l are the dimensions of the glued joint in mm.

#### 2.4.7. Bending Strength of Laminates

To determine the bending strength, the three-point bending test method was chosen. It was conducted using a TT 2850 universal testing machine (TIRA, Schalkau, Germany), according to ČSN 49 0115 [19]. The distance between the supports (l) was set to 240 mm. Four samples from each variant of laminate (290 × 40 × 7 mm) were tested. Modulus of rupture (MOR) in MPa was determined as follows:

(4) $$\mathrm{MOR}=\frac{3\cdot F_{\max}\cdot l}{2\cdot b\cdot t^{2}}\;,$$

where F_max_ is the maximum force achieved on the sample in N, l is the distance between supports (240 mm), b is the width, and t is the thickness of the sample in mm.

#### 2.4.8. Impact Bending Strength of Laminates

The determination of impact bending strength perpendicular to the plane of the plate was measured on an Impact 450 MPX-J2 Charpy hammer (INSTRON, Norwood, MA, USA). Ten samples (70 × 10 × 7 mm) from each variant were tested. The impact bending strength (IBS) in J·cm^−2^ was calculated according to the formula

(5) $$\mathrm{IBS}=\frac{W}{b\cdot t}\;,$$

where W is the work used to punch through the specimen in J, b is the width, and t is the thickness of the test specimen in cm^2^.

#### 2.4.9. Hardness of the Laminates

The hardness of the laminates was measured using the Brinell method on a DuraVision EMDlyV 30 universal hardness tester (EMCO-TEST Prüfmaschinen, Kuchl, Austria). A total of 15 measurements was made from each variant, when a steel ball with a diameter of 10 mm was pressed into the laminate with a stable force of 1000 N. Brinell hardness was evaluated automatically by the machine.

#### 2.4.10. Statistical Analysis

The measured data were characterised using descriptive statistics (arithmetic mean, minimum, maximum, and standard deviation). To determine the statistical significance of the differences between the individual variants, an analysis of variance (ANOVA) followed by a post hoc test (Tukey’s HSD test) was performed. These statistical tests were always performed at a significance level of α = 0.05 and using the Statistica program. In the Results and Discussion chapter, a graphical representation of the results is presented, where the vertical columns in the graphs represent 95% confidence intervals.

## 3. Results and Discussion

### 3.1. FTIR

**Figure 3:**
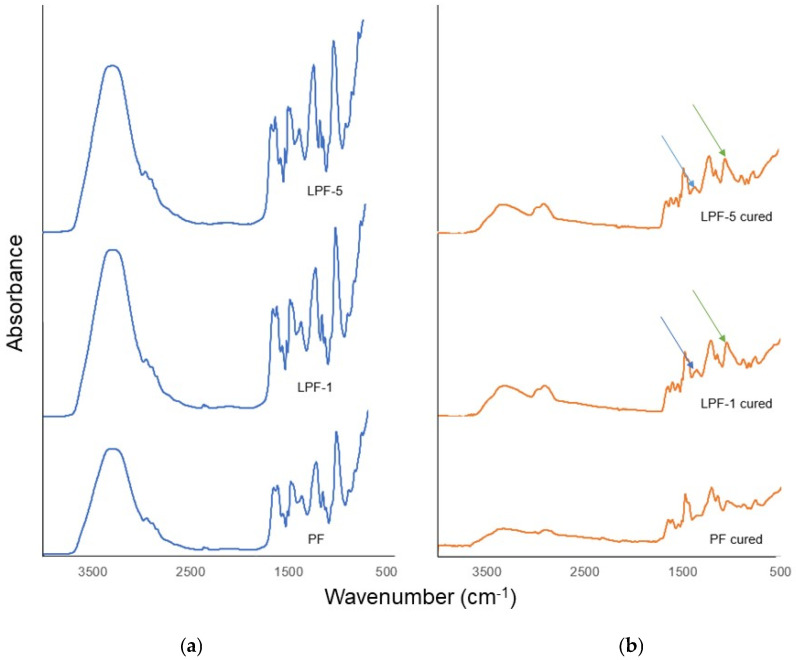
FTIR spectra of uncured (**a**) and cured (**b**) resins. Note: deformation vibration of bonds –CH of the aromatic ring (green arrow); stretching vibration of C–O (blue arrow).

Figure 3 shows that there is broadband in the region of 3000–3600 cm^−1^, which might be due to phenolic methylol hydroxyl (–OH) vibrations that are characteristic of PF resin. All uncured resins show the bands at 1630 and 1600 cm^−1^ that are ascribed to the aromatic carbon double-bond stretching vibration and the band at 1510 cm^−1^ that might be assigned to the phenolic ring substituted at the ortho- and para-positions. A band near 1200 cm^−1^ appears in the spectrum due to the stretching vibration of the phenol-O group and the intense band at 1006 cm^−1^ can be ascribed to the stretching vibrations of C–O–CH_3_ groups. The absorbance signals at wavenumbers 758, 800, and 875 cm^−1^ correspond to the number of adjacent hydrogens on the phenolic aromatic ring [20].

The curing of resins results in a significant decrease in the intensity of measured spectra (Figure 3), which was manifested mainly by the disappearance of the band ascribed to stretching vibrations of C–O–CH_3_ groups; however, the presence of the band characteristic for deformation vibration of bonds –CH of the aromatic ring in the wavenumber range 1045–1030 cm^−1^ is visible in the spectra of LPF resins (green arrow in Figure 3). One interpretation of the change observed could be that it is caused by the progressive polycondensation process, which leads to the release of –OH groups and the reactivation of C=O groups from formaldehyde. These C=O groups then react with the hydroxyl groups (–OH) present in the aromatic rings. The –CH2– methylene groups, however, remain unchanged in the resin structure between the phenolic rings during the curing process. Additionally, the observed decrease in the intensity of bands appearing in the range of 1270–1300 cm^−1^ may be a result of the stretching vibration of C–O of LPF resins weakening systematically (blue arrow in Figure 3).

### 3.2. Chemical Analysis of Resins

Table 2 and Table 3 show the general properties of the resin (PF, LPF-1, and LPF-5) and its main chemical components, specifically tannin, phenol, and formaldehyde.

Table 2 and Table 3 show the properties of the resin samples. It can be seen from these tables that as the substitution rate of the LPF resin increases, the density of the LPF resin increases accordingly. Because the molecular weight of phenol is lower than the molecular weight of lignin, we increased the molecular weight and internal frictional resistance of the resin by adding lignin. The pH value of LPF resins is the same for all types of resin; this is caused by the type of kraft lignin used, as it has been neutralised after precipitation with acid so it has a slightly acidic pH. The free formaldehyde content is not as high, and the reactivity of lignin is not as high as that of phenol. In the production process, a reaction between formaldehyde and lignin/phenol may have occurred, and a certain amount of unreacted formaldehyde may result in a decrease or increase in formaldehyde content, as well as solids content. However, solids content is around 35%, and free formaldehyde is lower than 0.06%, which conforms to the GB/T14074-2006 standard.

Chen et al. [21], dealing with the production of nanolignin-phenol-formaldehyde resin, report a pH value more fundamental than the resin we produced, which is apparently due to the more alkaline lignin and possibly a different type of original phenol-formaldehyde resin. The solids and formaldehyde content set values are almost identical to ours. Furthermore, regarding density, the results are slightly lower at around 1100–1200 kg·m^−3^.

However, Younesi-Kordkheili and Pizzi [22], comparing modification methods on the properties of lignin-phenol-formaldehyde resin, achieved almost the same density values, namely around 1220 kg·m^−3^. However, the solid content of the produced resins was higher at 60%. A similar density value (ca. 1230 kg·m^−3^) was also achieved in the case of work dealing with maleic and phenol-formaldehyde lignin [23].

In addition to the solid content and pH, Jin et al. [24] determined the phenol and formaldehyde content and achieved higher values for phenol and similar values for formaldehyde. However, all the values we measured also comply with GB/T 14732-2006.

### 3.3. Moisture Resistance of Laminates

**Figure 4:**
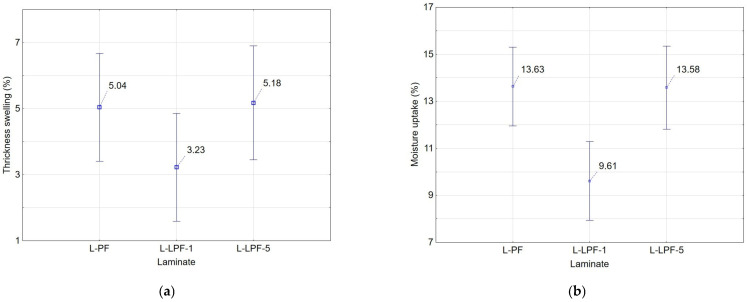
Influence of resin on (**a**) thickness swelling, (**b**) moisture uptake.

From Figure 4 it can be observed that the variant L-LPF-1 was significantly more resistant to thickness swelling and moisture absorption (TS 3.23%; MU 9.61%). The L-LPF-5 variant showed better thickness swelling results (5.18%) than the L-PF laminate (5.66%). The MU values were essentially identical for the L-PF and L-LPF-5 variants (13.63% and 13.58%, respectively). From the results, it can be said that the addition of 1% lignin to PF resin had a positive effect on TS and MU. This is probably due to the hydrophobic character of sulphated lignin [9], which would clearly be a positive factor in the utilisation of lignin in the production of phenolic resins. For laminates with the addition of 5% lignin, a significant deterioration can be observed compared to the L-LPF-1 variant, which may be due to the lower homogeneity of the internal structure of this variant.

### 3.4. Vertical Density Profile of Laminates

**Figure 5:**
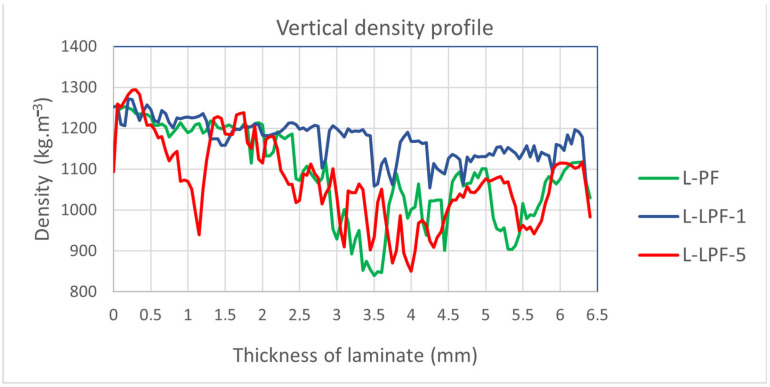
Vertical density profile of produced laminates.

When comparing VDP curves of individual laminate variants in Figure 5, it can be stated that the lowest variance is shown using the VDP curve of the L-LPF-1 variant, with a density ranging from 1250 to 1050 kg·m^−3^. This result confirms the homogeneity of the structure of this variant of the laminate, i.e., the results associated with this homogeneity. For the L-PF and L-LPF-5 variants, there is a significant difference in density on the surface and inside the tested laminates. This is caused by small delaminations inside the structure, which already occurred during the pressing of these laminates. However, none of the tested variants met the standard requirement for HPL density ρ ≥ 1.350 kg·m^−3^ (EN 438-1 [25]), which was caused by the low pressure applied during pressing of the laminates.

### 3.5. Scanning Electron Microscopy

**Figure 6:**
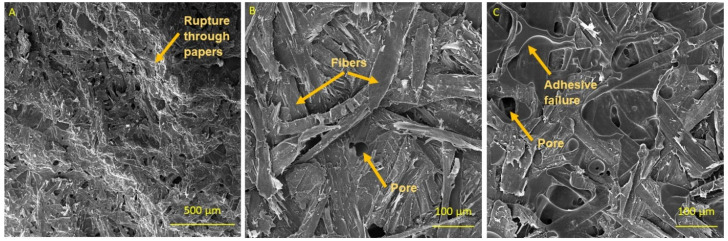
SEM of ruptured samples showing the character of bond rupture: (**A**) L-LPF-1 variant, magnification 150×; (**B**) L-LPF-1 variant, magnification 500×; (**C**) L-LPF-5 variant, magnification 500×.

Figure 6A presents rupture of L-LPF-1 laminate where a rupture through several layers of paper can be seen. The detailed figure of the sample from the same variant is shown in Figure 6B, where wood fibres of the paper are visible. In Figure 6B, cohesive failures are predominant, whereas in Figure 6C (rupture of L-LPF-5 laminate) adhesive failures are predominant. This corresponds to results of bending strength tests since the variant with highest amount of lignin filler (L-LPF-5) reached the lowest values of the MOR. SEM analysis also showed pores present in the laminates (marked with the arrows in Figure 6B,C). These kind of pores are formed in HPL based on PF resins during pressing when resin is cured and are a common phenomenon of laminates [15].

### 3.6. Mechanical Properties of Laminates

Taverna et al. [26] reported in their study that the structure of the cured resin (e.g., lignin characteristics, lignin content, and degree of curing), the type, orientation, and number of paper sheets in the laminate and the mutual interaction between paper and resin affect the mechanical properties of HPL. An optimal ratio of lignosulfonate: phenol was 10:90. The dry matter of such resin was 49.2%, and the density 1.06 g/mL [26]. The influence of resin interaction on the resulting mechanical properties of high-pressure laminates is confirmed by Thébault et al. [16], where they state that resins with a tendency to deposit residues on the surface of the laminate (i.e., liquids with a long penetration time) subsequently resulted in an inhomogeneous distribution of the resin in HPL, thereby reducing the mechanical properties. The lowest tensile strength of 63.2 MPa was achieved by laminate produced with a LPF resin where 50% of phenol was substituted with lignin, whereas the highest tensile strength of 133 MPa was reached using laminate made from pure PF resin.

Based on these findings, it can be expected that the LPF-5 resin, which showed a significant increase in viscosity during its production and subsequent impregnation, will exhibit lower mechanical properties than the reference PF resin and the LPF-1 resin.

**Figure 7:**
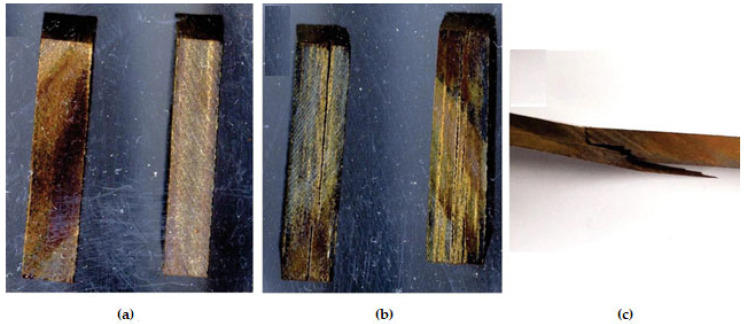
Cross section of L-LPF-1 laminate without delamination (**a**); cross section of L-LPF-5 laminate with the appearance of delamination (**b**); fracture structure of L-LPF-1 after bending strength test (**c**).

The structure of the manufactured laminates was already affected during their production. The critical point was the cooling of the manufactured laminates after the pressing process. In the industrial case, cooling of laminates is carried out during the pressing cycle using a coolant [1]. The cooling of laminates during the pressing cycle prevents the penetration of vapours through the surface of the boards [27]. In this study, it was not possible to introduce cooling during pressing, which resulted in minor internal and surface delamination in the final HPL (see Figure 7a,b).

#### 3.6.1. Tensile Shear Strength of the Glued Joint

**Figure 8:**
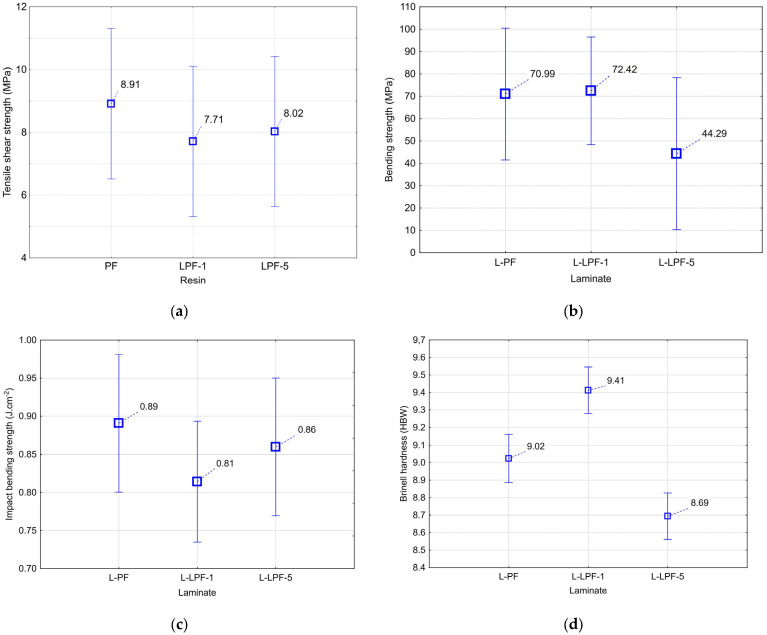
Influence of resin on (**a**) tensile shear strength, (**b**) bending strength, (**c**) impact bending strength, (**d**) Brinell hardness. Note: vertical bars represent 95% confidence intervals; *p*-values are presented in Table 4.

As can be seen in Figure 8a, the commercial PF resin achieved the highest tensile shear strength (8.91 MPa). All samples bonded with commercial PF resin had a joint failure in the wood. Adding lignin to the commercial resin resulted in a decrease in tensile shear strength. LPF-1 resin showed lower strength (7.71 MPa) than LPF-5 resin with higher lignin addition (8.02 MPa). For both resins enriched with lignin, joint failure occurred simultaneously in both the wood and the glue. The reduction in tensile shear strength can be attributed to less than perfect dissolution of lignin macromolecules in the PF resin. This leads to an increase in the molecular weight of the adhesive, which improves cohesive strength but has a negative impact on adhesion. Undissolved lignin molecules can also act as impurities when their concentration near the glued joint creates a weak connection to the substrate and therefore lower mechanical properties [28].

#### 3.6.2. Bending Strength of Laminates

The bending strength results (Figure 8b) show that adding 1% lignin to the PF resin resulted in a slight increase in the MOR (72.42 MPa) for L-LPF-1 laminates compared to L-PF laminates (70.99 MPa). Conversely, a significant reduction in the MOR (44.29 MPa) was observed for L-LPF-5 laminates. The results show that the addition of lignin to commercial PF in values of 5% or more has a negative effect on the MOR of laminates.

In the case of L-LPF-1 laminates, a highly homogeneous structure was also observed at the point of fracture (see Figure 6A and Figure 7c), where the fracture occurred at the point of application of force over a relatively short length. In contrast, with the other variants, sample breakage was observed in one or more layers of paper; this fracture penetrated a considerable length of the laminate sample. A similar fracture of L-LPF-1 samples was achieved by Thébault et al. [16]; in this case it was, however, an undesirable phenomenon compared to the mere bending of the samples without major damage. In the work of Thébault et al. [16], laminates in which phenol was replaced in the resin with different contents of lignin did not achieve comparable flexural strength to the reference sample.

#### 3.6.3. Impact Bending Strength of Laminates

The highest IBS was achieved using the L-PF laminate variant (0.89 J∙cm^−2^) (Figure 8c). The worst results measured were for L-LPF-1 laminates (0.81 J∙cm^−2^). The L-LPF-5 variant showed toughness of 0.86 J∙cm^−2^, where, in contrast to MOR, the addition of a higher amount of lignin did not significantly reduce the measured property. The opposite effect was achieved using the L-LPF-1 variant, where it can be concluded from the results that these laminates were very strong, but at the same time very fragile.

A similar fracture effect was observed for all samples, where a fracture occurred on the side of the applied force, while on the other side a part of the laminate broke off or even delaminated the sample in a certain layer. A similar refraction effect was observed by Taverna et al. [13].

#### 3.6.4. Hardness of the Laminate Surface

Figure 8d shows that the highest Brinell hardness was exhibited by the L-LPF-1 variant laminate (9.41). This high hardness is probably the result of the very homogeneous structure of the tested samples. The reference laminate L-PF showed a Brinell hardness of 9.02. The L-LPF-5 variant fared the worst, with a Brinell hardness of 8.69. For the variants L-PF and L-LPF-5, the decrease in hardness of the laminates was probably caused by local delamination inside the test samples and thus by a not completely homogeneous internal structure.

## 4. Conclusions

The presented research showed that even adding only 1% lignin to the PF resin affects the resulting HPL composite. In this study, kraft lignin was used for the modification of PF resin. Kraft lignin is less reactive than lignosulfonate; however, substantially more kraft lignin is produced than lignosulfonate worldwide. The selected ratios of lignin addition to PF resin were 1 and 5% (solid to solid). The results showed that the addition of 1% lignin to PF resin resulted in better properties than the addition of 5% lignin to PF resin. Moreover, laminates with 1% lignin exhibited better properties (bending strength, Brinell hardness, moisture uptake, and thickness swelling) than reference samples produced with commercial PF resin. The addition of 5% lignin to PF resin is not recommended, since the produced HPL had worse properties than HPL manufactured with reference PF resin and, in addition, delamination occurred in the L-LPF-5 variant. It can be concluded that the addition of lignin in pulverised form to commercial resin in small amounts is a promising direction since produced kraft lignin does not need to be burned.

## Figures and Tables

**Table 1:** Variants of resins and laminates.

Resin Variant	Description	Laminate Variant	Description
PF	Commercial PF resin	L-PF	Laminate bonded with commercial PF resin
LPF-1	Commercial PF resin filled with 1% of lignin	L-LPF-1	Laminate bonded with commercial LPF-1 resin
LPF-5	Commercial PF resin filled with 5% of lignin	L-LPF-5	Laminate bonded with commercial LPF-5 resin

**Table 2:** Chemical analysis of resins.

Resin/Chemical Component	Phenol, %	Formaldehyde, %	Tannin, %
PF	0.004 (0.00)	0.003 (0.00)	0.000 (0.00)
LPF-1	0.044 (0.08)	0.017 (0.01)	0.270 (0.00)
LPF-5	0.168 (0.03)	0.065 (0.02)	1.000 (0.00)
GB/T 14732-2006	<6.000	<0.300	–

Standard deviation values are in parenthesis.

**Table 3:** General properties of resins at T = 21 °C.

Resin/General Properties	pH	Solid Content, %	Density, kg·m^−3^
PF	8.72 (0.03)	79.66 (0.17)	1247.8 (0.12)
LPF-1	9.08 (0.07)	35.64 (0.23)	1222.4 (0.35)
LPF-5	8.62 (0.04)	36.07 (0.33)	1224.1 (0.28)
GB/T 14732-2006	>7.00	>35.00	–

Standard deviation values are in parenthesis.

**Table 4:** The *p*-values of differences presented in Figure 8.

Tensile Shear Strength			
Sample	L-PF	L-LPF-1	L-LPF-5
L-PF		0.74803	0.852602
L-LPF-1	0.74803		0.980253
L-LPF-5	0.852602	0.980253	
Impact bending strength			
Sample	L-PF	L-LPF-1	L-LPF-5
L-PF		0.404424	0.873932
L-LPF-1	0.404424		0.720273
L-LPF-5	0.873932	0.720273	
Bending strength			
Sample	L-PF	L-LPF-1	L-LPF-5
L-PF		0.996234	0.416119
L-LPF-1	0.996234		0.330311
L-LPF-5	0.416119	0.330311	
Brinell hardness			
Sample	L-PF	L-LPF-1	L-LPF-5
L-PF		0.000611	0.003374
L-LPF-1	0.000611		0.00013
L-LPF-5	0.003374	0.00013	

## Data Availability

The data presented in this study are available on request from the corresponding author. The data are not publicly available due to unfinished research.

## Nomenclature

HPL	High-pressure laminate
PF	Phenol-formaldehyde
P	Phenol
F	Formaldehyde
LPF-1	Commercial PF resin filled by 1% of lignin
LPF-5	Commercial PF resin filled by 5% of lignin
L-PF	Laminate bonded with commercial PF resin
L-LPF-1	Laminate bonded with commercial LPF-1 resin
L-LPF-5	Laminate bonded with commercial LPF-5 resin
FTIR	Fourier-transform infrared spectroscopy
ATR mode	Attenuated Total Reflectance mode
TS	Thickness swelling
MU	Moisture uptake
VDP	Vertical density profile
SEM	Scanning electron microscopy
MOR	Modulus of rupture
TSS	Tensile shear strength
IBS	Impact bending strength
